# The Five Basic Human Senses Evoke Electrodermal Activity

**DOI:** 10.3390/s23198181

**Published:** 2023-09-29

**Authors:** Dindar S. Bari, Mohammed Noor S. Rammoo, Haval Y. Y. Aldosky, Mohammed K. Jaqsi, Ørjan G. Martinsen

**Affiliations:** 1 Department of Physics, Faculty of Science, University of Zakho, Zakho 42002, Kurdistan Region, Iraq; dindar.bari@uoz.edu.krd (D.S.B.); mohammed.noor@uoz.edu.krd (M.N.S.R.);; 2 Department of Physics, College of Science, University of Duhok, Duhok 99454, Kurdistan Region, Iraq; yacoobaldosky@uod.ac; 3 Department of Clinical and Biomedical Engineering, Oslo University Hospital, 0424 Oslo, Norway; 4 Department of Physics, University of Oslo, Sem Sælands vei 24, 0371 Oslo, Norway

**Keywords:** electrodermal activity, electrodermal responses, senses, skin, sweat

## Abstract

Electrodermal activity (EDA) usually relates to variations in the electrical properties of palmar or plantar skin sites. EDA responses, namely skin conductance responses (SCRs), skin potential responses (SPRs) and skin susceptance responses (SSRs) are shown to be sensitive indexes of sympathetic nervous system activation and are studied in many research projects. However, the association between EDA responses and the five basic human senses has not been investigated yet. Our study aimed to explore the relationship between the three EDA responses (SCRs, SSRs and SPRs) and the five basic human senses. These three EDA responses were measured simultaneously at the same skin site on each of the 38 volunteers. The tested five senses were sight, hearing, touch, taste and smell. The results showed that the different tested senses led to different degrees of EDA responses due to activation of the sympathetic nervous system and corresponding secretion of sweat. Although a controlled study on the degree of EDA as a function of the strength of each stimulus was not performed, we noted that the largest EDA responses were typically associated with the smell sense test. We conclude that EDA responses could be utilized as measures for examining the sensitivity of the human senses. Hence, EDA devices may have important roles in sensory systems for future clinical applications.

## 1. Introduction

Electrodermal activity (EDA) refers to all active and passive electrical properties observed in the skin and its appendages, but the term is usually associated with the changes in these parameters that are due to activity in the sympathetic nervous system [1]. The EDA parameters, skin potential (SP), skin conductance (SC) and skin susceptance (SS) will typically rapidly increase with sweating since sweat mostly contains electrolytes and water. Sweating leads to the creation of ionic pathways in the dry (highly resistive) outermost stratum corneum layer of the skin and hence leads to swift changes in EDA [2]. EDA signals are subsequently recovered due to sweat reabsorption or duct closures. Therefore, changes in EDA follow sweating since the sweat ducts are filled and emptied and stratum corneum hydration consequently varies. EDA signals are significantly stronger in the palmar and plantar skin sites (in the hands and under the feet) due to the sympathetic innervation and extensive number of sweat glands in these regions [3,4]. In addition to their main role in thermoregulation, sweat glands (especially eccrine glands) also respond to psychological situations [5]. Sweat is then controlled by the sympathetic part of the autonomic nervous system and hence EDA could be an important physiological indicator of sympathetic arousal with important implications for future medical biosensing [1].

Studies of the electrical properties and features of human skin have been carried out for more than a century. In 1879, Vigouroux found that the electrodermal response is related to psychological factors in which skin resistance involuntarily changes following certain external stimuli. Fere in 1888 and Tarchanoff in 1889 performed pioneering studies of EDA. Fere observed a reduction in the skin’s electrical resistance as a result of sensory or emotional stimuli when using externally applied direct current. The method used by Fere is an exosomatic method, as a small current is applied to the skin from an external source, and it is often called the Fere effect (phenomenon) [6]. On the other hand, Tarchanoff noticed alterations in skin’s electrical potential related to sensory stimulation without utilizing any external electrical current source [7]. The method employed by Tarchanoff is an endosomatic method and is often termed the Tarchanoff effect (phenomenon), as EDA is measured without using an external current source. Therefore, in the Fere method, changes in skin resistance to the passage of a weak electric current are recorded, while in Tarchanoff’s method, a weak current actually generated by the body is measured. Later, Tarchanoff showed that, in addition to physical stimuli, mental activity also led to changes in SPRs. Among other eminent researchers who contributed to the study of the electrodermal response were Georg Sticker (1860–1960) and Otto Veraguth (1870–1944) [6]. Historically, several terms are used to illustrate EDA phenomena, such as psychogalvanic reflex and galvanic skin response, which was named by Luigi Galvani (1737–1798), who first discovered animal electricity [6]. However, in 1966 electrodermal activity (EDA) was suggested as a common term for all electrical phenomena in the skin [1].

Although the main role of sweat glands is body temperature regulation, those located in the palms and soles are also responsive to psychological stimuli and emotionally induce sweating. They are innervated by the sympathetic nervous system, mainly sudomotor nerves, and regulated by acetylcholine and noradrenaline neurotransmitters. For EDA recording, catecholamines such as noradrenaline have a main role because these neurotransmitters, in addition to controlling sweat glands, play an important role, particularly in emotionally induced sweating [8]. Various cerebral structures and pathways are involved in sweat gland activation and hence EDA signal production, including hypothalamic control, contralateral and basal ganglia, reticular formation and different cortical areas.

EDA is generally separated into two components. The first is the tonic component which varies slowly over time. This component is shown to represent different ambient conditions, particularly humidity and temperature which contribute to the sweat glands filling in the absence of sudomotor nerve activity [1]. Tonic EDA can be skin potential level (SPL), skin conductance level (SCL) and skin susceptance level (SSL). The second is the phasic component, which is characterized by a rapid increase to a peak value and a slower recovery to the baseline as a direct result of a sudomotor burst. The balance between the intraductal pressure and the subsequent hydraulic pressure of the stratum corneum controls the pore opening and closure mechanism that affects the phasic component [9]. Phasic EDA is represented by skin potential responses (SPRs), skin conductance responses (SCRs) and skin susceptance responses (SSRs).

The recording methods of EDA are mainly categorized as exosomatic or endosomatic. In the exosomatic approach, an external source of either an AC or DC voltage is typically applied to the skin and the corresponding current is measured. If an AC source is used, skin admittance can be measured from which skin conductance (SC) and skin susceptance (SS) are obtained, while only SC can be measured if the applied source is DC. In the endosomatic approach, the potential produced by the skin is recorded, so no external energy source is needed. Although the endosomatic method is simpler than the exosomatic method, endosomatic SPRs show complex waveforms such as positive and negative monophasic, biphasic, or even triphasic waves, which complicate the analysis of data. Exosomatic SCRs are only monophasic; therefore, exosomatic DC recording by using a controlled voltage is traditionally the most widely employed [1].

EDA has instigated several clinical applications due to ease of use, noninvasive data collection and high sensitivity to sweat activity. EDA is used as a tool for stress assessment [10,11], sleepiness, pain monitoring, psychiatric disorder assessment, nerve blocking assessment [12,13], skin moisture assessment, depression, diagnosis of epilepsy and various neurological disorders [14].

Despite an increasing volume of EDA research over the last few decades, there are no studies on the relationship between the five basic human senses and the three EDA parameters, except for a few studies which are concerned with only one EDA parameter (SC) and some of the human senses (i.e., not all of the five basic human senses together) [2,15,16,17,18,19]. Human sensing is a means of receiving information from the surrounding environment and converting it to an electrical signal that is processed in the brain. Humans mainly understand and perceive the surrounding environment (world) through the five senses, namely gustation (taste), olfaction (smell), audition (hearing), vision (sight) and touch. These senses are the channels that feed the brain with information to help us build a picture of the complicated world around us.

Using a novel method for simultaneous measurement of SC, SP and SS developed in our group [2], this study intended to comprehensively investigate the association between the five basic human senses and these three EDA recordings. To the authors’ knowledge, this study is the first to thoroughly investigate the relationship between these three parameters of EDA and human senses, taking into account all five basic senses.

The rest of the paper is structured as follows. In Section 2, related works are presented. In Section 3, the employed measurement setup, experimental protocol, data and statistical analysis are described. In Section 4, the obtained results are presented. In Section 5, discussion of the obtained results is provided. Finally, in Section 6, conclusions are drawn.

## 2. Related Works

As mentioned above, few studies in the literature are focused on the relationship between EDA and emotional or arousal senses, which are concerned with only one EDA parameter (SC) and some of the human senses (i.e., not all five basic senses together). For example, Gatti et al. [15] investigated SC responses to visual, auditory and haptic stimuli. Iadarola et al. [16] studied the effect of three different acoustic stimuli on SCR. The researchers found a higher number of SC peaks due to unpleasant and neutral stimuli compared to those from a pleasant stimulus. Greco et al. [17] proposed a convex optimization-based EDA (SC measuring) system to detect SC responses due to various arousal and valence levels. The authors reported that their system could attain good accuracy in recognition of arousal and valence dimensions. Zhu et al. [18] used SC to observe healthy persons and individuals with depression and found that SC is better suited for identifying healthy than depressed states. Iadarola et al. [19] developed an approach based on Compressed Sensing for detecting SC peaks. They showed that the proposed approach was better than the existing toolboxes such as Ledalab for automatic identification of the correct number of SC peaks. Bari et al. [2] investigated the effects of some emotional and arousal stimuli on SCRs, SPRs and SSRs and obtained different peak values of SCRs, SPRs and SSRs depending on the type of stimuli.

The above cited studies are focused on recording only one EDA response parameter or one or two senses as a result of some psychological stimuli, without directly examining their relationship to all the five basic senses. In this paper, three EDA (SCRs, SSRs and SPRs) parameters are recorded with the aim of investigating their correlation with the five senses.

## 3. Materials and Methods

A computer-based system for measurements of EDA parameters (SC, SS and SP) simultaneously at the same skin site was used. The system was composed of a small front-end electronic circuit, which was connected to a computer running software written in LabVIEW, v. 14^®^ via a data acquisition (DAQ) card from National Instruments [2]. To record the three EDA parameters simultaneously, the three-electrode measurement setup was utilized, and the type of electrode used was Kendall Kittycat 1050NPSM Ag/AgCl solid gel ECG neonatal electrodes.

The set-up was the same as described in [2], where a three-electrode system was used consisting of one measuring electrode, one reference electrode, and a current-sink electrode. The DC voltage between the measuring electrode and the reference electrode (which is placed at an electrodermally inactive skin site such as the apex of the elbow) is utilized for SP measurement. At the same time, both the current sink electrode (which is placed on the underarm), and the reference electrode (which is placed on an electrodermally active skin site such as the hypothenar site of the palm), are used to provide SC and SS measurements beneath the measuring electrode.

A Howland current pump was used to provide the AC current. A 200 mV voltage was produced by the DAQ system and fed to the Howland circuit. The Howland circuit in turn delivered about 20 μA AC (proportional to the input voltage) to the skin through the measuring electrode. The DAQ card received the analog signals back from the skin via the electronic front-end electronic box and converted them to digital form. The digitized signals were then processed by differentiation in the LabVIEW software and separated into a DC component for SP and an AC component for SC and SS by using a conventional lock-in technique (phase-sensitive rectification). The lock-in amplifier (synchronous rectifier) also functioned as a band pass filter which removed or reduced any noise in the measured signals.

### 3.1. Experimental Protocol

Experiments were carried out on 38 healthy Caucasian participants (19 male and 19 female) with an age range between 18 and 44 (mean 25.71 years). Participants were recruited from the population of undergraduate students as well as academic staff at the University of Zakho. They had normal vision and were devoid of any past or present neurological or mental disorders, medical treatments that might modify emotional processes, and other conditions. Participants who were easily upset by music and had excessive or diminished sensitivity to tastes or smells as a result of COVID-19 infection were excluded from the study. Before starting the experiment, the nature and aim of the study were explained to all participants and written informed consent was obtained from all of them. During measurements of EDA, all participants were sitting comfortably in a chair in a calm room and away from vibration-prone areas at normal room temperature (22–23 °C). All subjects were asked to relax, to remain awake, and to avoid bodily movement. Any speaking was not allowed for participants during the whole session of data collection. Hence, the EDA signals were recorded in a calm indoor environment free of noise.

**Figure 1:**
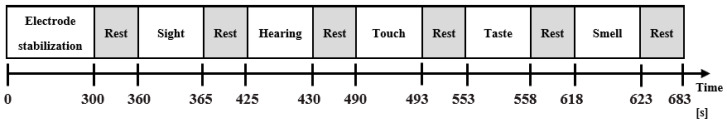
Timeline and sequence of tests.

After the application of electrodes to one hand of each of the participants, at least five minutes were required for their stabilization prior to starting the measurements of EDA. Then to test the senses of the participants, they were subjected to five sensory tests, which were vision (sight; looking at a positive nature photo for 5 s), audition (hearing; listening to relaxing (soothing) piano music for 5 s), touch (mild (light) clap on the participant’s shoulder for 3 s), gustation (taste; drinking a spoon of lemon (sour flavor) water for 5 s) and olfaction (smell; inhaling a pleasant odor for 5 s) as shown in Figure 1. Before and after each sensory test, there was a rest time of 60 s. It should be noted that after the tests all participants were asked whether they had, or had not, a feeling of sense for all the separate sensory tests.

### 3.2. Data and Statistical Analysis

Measured EDA signals were analyzed by obtaining some specific scores from the signals. Scores were computed as illustrated in Bari et al. [2]. The first response following any sense at the specified time (duration of stimulus) was chosen and analyzed. The scores were:

- amplitude of the skin potential responses (SPRs_Amp)
- amplitude of the skin conductance responses (SCRs_Amp)
- amplitude of the skin susceptance responses (SSRs_Amp)
- time from onset of SCRs to peak SCRs (SCRs_Tris)
- skin potential relative early turn (SPRET).

Table 1 provides a comprehensive list of all the parameters that were taken from the EDA responses and used in the data analysis.

The SCR_Amp, SPR_Amp and SSR_Amp were obtained from the difference between the response maximums (peaks) and the onsets (SCL, SPL and SSL) of SCRs, SPRs and SSRs, respectively. The SCRs_Tris was calculated from the time difference between onset and SCRs peaks. SPRET was calculated by subtracting the SCRs peak time from the SPRs peak time, dividing the result by the SCRs onset time to SCRs peak time, and multiplying the result by 100% [20].

To statistically assess the variations in the recorded EDA responses during the five sensory tests, a one-way repeated analysis of variation (ANOVA) was used. ANOVA was found to be the best fitted statistical test for this study, since several measurements were done on each participant. Also, it has a high statistical power, and fewer participants are required. The ANOVA test was computed by comparing every EDA score for each participant (dependent variable) for different stimuli (i.e., different independent groups/variables). This was done by using a general linear model/repeated measures in SPSS. Also, to compare groups against each other, post hoc multiple pairwise comparisons employing Sidak correction were conducted. The statistical analyses were done using IBM SPSS Statistics.

**Figure 2:**
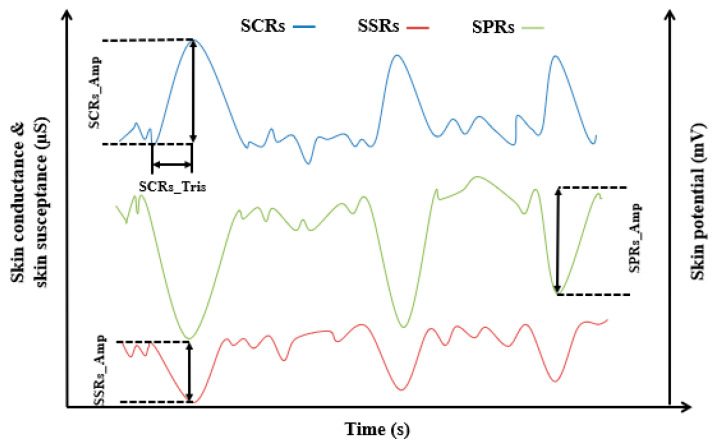
Examples showing SCRs, SPRs and SSRs.

Figure 2 shows how onsets and peaks from SCRs, SSRs and SPRs were specified and EDA scores obtained.

## 4. Results

### 4.1. Amplitude of EDA Responses

**Figure 3:**
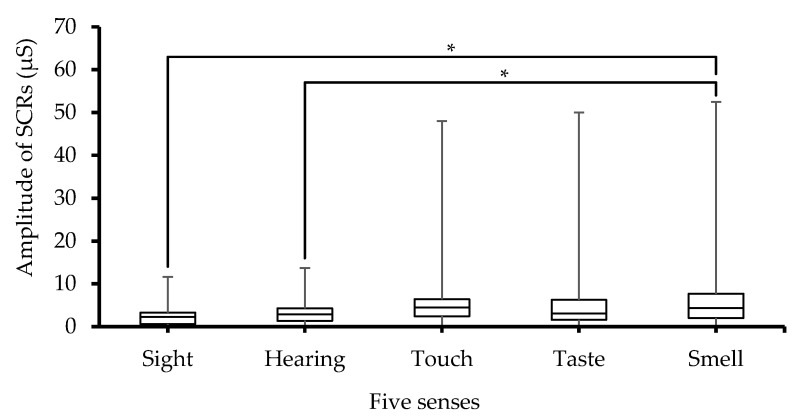
Box-plot with medians, quartiles, and the min and max as whiskers, showing SCRs_Amp in relation to the five senses, * *p* < 0.05.

Figure 3 shows the median value of SCRs_Amp as a function of activation of each of the five senses. The stimulus of the different senses leads to different SCRs_Amp values. The ANOVA test also detected significant (*p* < 0.001) differences between the groups (the five senses). Moreover, post hoc pairwise multiple comparison tests revealed significant (*p* < 0.05) differences between the smell sense on one hand and sight and hearing senses on the other hand, as indicated in the figure. The error bars shown in Figure 3 are the minimum and maximum of the data obtained from *n* = 38 participants. The obtained error level is not due to uncertainty in the recorded data, but it is rather due to individual differences as the SCRs_Amp data are gathered from a group of different participants. Furthermore, the statistical analysis with ANOVA also confirmed between-subjects differences, which were significant (*p* < 0.001).

**Figure 4:**
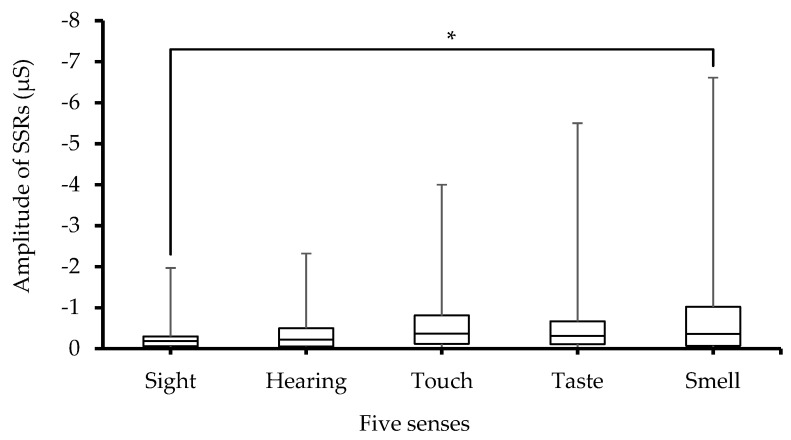
Box-plot with medians, quartiles, and the min and max as whiskers, showing SSRs_Amp in relation to the five senses, * *p* < 0.05.

Variations in SSRs_Amp values following stimulus of the five senses are depicted in Figure 4. Statistical analysis of SSRs_Amp data showed significant (*p* < 0.001) differences between the five sense groups. Moreover, it can be seen that the maximum values (indicated through whiskers) of SSRs_ Amp with respect to taste and smell stimuli are larger than those for sight, hearing and touch, and reached the largest value for the smell stimulus. Furthermore, post hoc pairwise multiple comparisons yielded a significant (*p* < 0.05) difference between the smell and sight tests, while differences among other groups were non-significant. The large error bars seen in Figure 4 indicate that there are large between-subjects differences in SSRs_Amp, which were also corroborated via ANOVA tests and showed significant (*p* < 0.001) differences between subjects.

**Figure 5:**
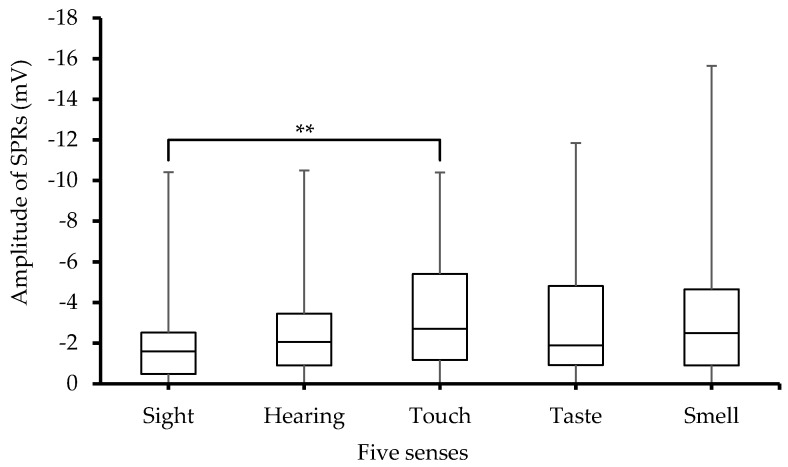
Box-plot with medians, quartiles, and the min and max as whiskers, showing SPRs_Amp in relation to the five senses, ** *p* < 0.005.

SPRs_Amp also behaved differently for the five senses. Furthermore, the ANOVA analysis revealed highly significant (*p* < 0.001) differences among the groups. Furthermore, Sidak post hoc pairwise multiple comparisons showed significant (*p* < 0.005) differences between the sight and touch senses. There were also significant (*p* < 0.001) differences between the subjects as indicated by the ANOVA analysis as well as large error bars seen in Figure 5.

### 4.2. Timing Components of the Responses

#### 4.2.1. Skin Potential Relative Early Turn

**Figure 6:**
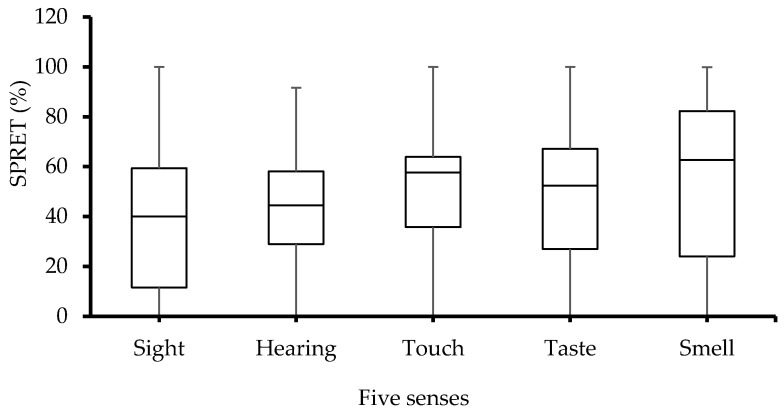
Box-plot with medians, quartiles, and the min and max as whiskers, showing SPRET in relation to the five senses.

The skin potential relative early turns (SPRET) were also significantly (*p* > 0.05) influenced by the five different senses as shown in Figure 6. However, post hoc pairwise multiple comparison indicated that none of the group differences (sense tests) were significant. Although the error bars on Figure 6 appear to be visually significant, the statistical analyses with ANOVA tests showed that the differences between subjects represented by those error bars were not statistically significant (*p* > 0.05).

#### 4.2.2. The Rise Time of Skin Conductance Responses

**Figure 7:**
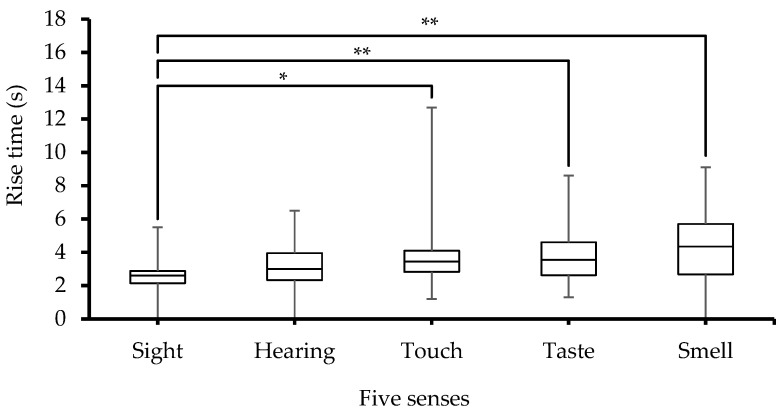
Box-plot with medians, quartiles, and the min and max as whiskers, showing SCRs_Tris in relation to the five senses, * *p* < 0.05 and ** *p* < 0.005.

ANOVA analysis on SCRs_Tris showed significant differences among the five groups (*p* < 0.01). Moreover, group comparison tests also revealed a significant (*p* < 0.05) and highly significant (*p* < 0.005) difference between sight on one hand and touch, taste and smell, on the other hand, as noted in Figure 7. Furthermore, both the ANOVA analysis and large error bars seen in Figure 7 point to significant between-subjects differences in SCRs_Tris.

## 5. Discussion

This work was conducted to study the possibility of changes in SC, SS and SP responses to stimulation of the five basic human senses. Generally, the obtained results demonstrated that activation of all five senses was able to elicit EDA responses in all participants (*n* = 38). The responsivity of the sympathetic nervous system to various senses causes different EDA responses, as measured by SP, SS and SC. Stimulation of the senses contributes to cognitive development and mental activity, which then leads to evoking EDA responses. Furthermore, other factors such as prolonged wakefulness can cause cognitive deterioration and delay responses, which can be detected by measuring EDA responses and other modalities [21]. EDA could also be evoked by pain anticipation and sensation as a submodality of somatic sensation and hence can be considered as a complex experience with cognitive aspects because it induces sympathetic responses [22]. However, although both sensation and anticipation activate the sympathetic system and consequently EDA responses, EDA responses generated with pain sensation are faster and have a greater amplitude than those obtained with pain anticipation [23]. Compared to the five basic senses investigated in this study, pain sensation differs from these five senses (sight, hearing, touch, taste and smell) because it is both a discriminative sensation and a graded emotional experience caused by intense or damaging stimuli, which might cause larger EDA responses. Moreover, many researchers have demonstrated that changes in EDA responses are highly correlated with changes in mental activity and cognitive tasks due to their sensitivity to changes in the sympathetic system. Therefore, EDA has been successfully used in various applications such as psychophysiological measurements for lie detection [1], assessing and predicting impaired cognitive performance due to prolonged wakefulness [24], etc. In this study, the magnitudes of the responses changed within subjects and also between the different kinds of senses. Furthermore, the EDA responses in general were larger for the smell sense compared to the rest of the senses. However, it should be noted that we did not make any attempt to adjust the intensities of the different stimuli to match one another.

Different senses cause variations in SCRs_Amp. The increase in SCRs_Amp from the smell test was significantly larger than that from the other senses. This could be caused by the intensity of the odor used, but it could also be related to the fact that the smell sense is the strongest human sense compared to the other senses due to the fast response of the olfactory receptor neurons that instantly send information directly to the brain [25]. Consequently, SCRs_Amp is increased following sweat duct filling due to arousal of the sympathetic nervous system. According to all EDA models, the SCRs_Amp is completely based on the sweat ducts in the skin. Moreover, once the sweat ducts are filled, SCRs_Amp is increased as a result of the reduction of skin resistance and the creation of ionic transport pathways in the stratum corneum [1,26].

Furthermore, the study results demonstrate that the SSRs_Amp is affected by the different senses. Like SCRs_Amp, the maximum value of the SSRs_Amp is associated with the smell sense. Moreover, an inspection of Figure 4 suggests that this EDA parameter is more affected by taste and smell stimuli than sight, hearing and touch stimuli. Variations in SSRs_Amp are due to alterations in skin moisturization because of sweat secretion (as a consequence of arousal of the sympathetic nervous system) since SSRs are highly associated with the moisturization of the stratum corneum [27]. In addition, in this study SSRs and the other EDA parameters are more influenced by the chemical stimuli (taste and smell, which are associated with chemoreceptors) than the physical ones (sight, hearing and touch, which are related to photoreception and mechanoreceptors). High error bar values in SSRs (Figure 4) due to taste and smell stimuli are notable proof of individual variability among the sampled data points. This indicates that there is a significant interindividual variability in EDA responses to chemical stimuli (taste and smell). Therefore, a higher SSRs_Amp due to taste and smell stimuli, particularly the smell stimulus, means that the skin is more moisturized due to these stimuli as compared to sight, hearing and touch stimuli.

Similarly, a relationship (Figure 5) between SPRs_Amp and the five senses is observed. The highest SPRs_Amp is associated with the smell sense. The increase in SPRs_Amp can be explained by the poral valve and voltage-divider models of EDA [9,28]. According to the poral valve model, when the sweat ducts are partially filled the SPRs are negatively increased (more negative) since they provide more conductive pathways through the stratum corneum to the more negative epidermal skin potential. Based on the voltage-divider model, SPRs are increased because the conductive sweat improves contact with the negative potential in the pore.

Regarding the timing of the different components of the responses, both SPRET and SCRs_Tris show different values as a function of the five senses tests.

It is clear from the box-plot presented in Figure 6 that the highest SPRET percentage is obtained from the smell sense. Moreover, all SPRET values were between 0% and 100%, which means that no negative SPRET (i.e., SPRET < 0) is seen in line with [2,14]. As a result, 0% SPRET means that SPRs peaked at the same time as SCRs and a SPRET greater than 0% up to 100% means that SPRs peaked earlier than the SCR peak. In addition, SPRET values depend on the sweat duct filling, in which SPRET tends to be 100% once the sweat duct is already filled to its limp capacity before sweat secretion, and it will be 0% when an empty duct is filled without raising intraductal pressure [20].

The highest SCRs_Tris value is also associated with the smell sense, which is attributed to the high value of SCRs_Amp due to the same sense. Also, in another study, Bari et al. [2] showed that the longer SCRs_Tris is due to the higher SCRs_Amp as a long time is required by SCRs to reach their peaks.

### Limitations of the Study

The limitation of this study was that only one specific stimulus for each sense was chosen. In further studies, it would be interesting to involve various (from lower to stronger) levels of stimuli for each type of sense to explore whether different levels of stimuli give different EDA responses.

## 6. Conclusions

This study focused on investigating the relationship between three (SC, SS and SP) parameters of EDA and the five basic human senses. The findings from this study show that different EDA responses were associated with the different senses. Activation of all five senses was able to elicit EDA responses in different magnitudes depending on the type of stimuli, since it contributes to cognitive load and mental activity that are correlated with EDA responses. Therefore, the five fundamental senses of humans and cognitive load are connected. The amount of cognitive processing required to comprehend sensory information can vary depending on complexity, clarity and volume, which will eventually affect how much cognitive load a person experiences when engaging in various tasks and activities. In this study, EDA parameters were more influenced by the chemical stimuli (taste and smell) than the physical ones (sight, hearing and touch) for the levels of stimuli used in this study. The highest SCRs, SSRs and SPRs were generally due to the smell sense. Ultimately, this study was able to ensure the reliability and validity of the findings by utilizing a tailor-made instrument that can record EDA signals in a quiet, noise-free indoor setting and minimize the impact of ambient frequencies. EDA responses may be used as indexes for testing the effectiveness of the five main human senses. Therefore, EDA instruments may have important roles in future clinical applications.

## Figures and Tables

**Table 1:** Overview of all EDA scores obtained from the responses that were used in the analysis.

EDA Score	Description	Unit
SCRs_Amp	Amplitude of the skin conductance responses. Typically range from threshold (onset of the SCRs) to the peak (maximum) value within the SCRs. The SCRs are always positive monophasic responses.	μS
SSRs_Amp	Amplitude of the skin susceptance responses. Typically range from onset of the SSRs to the peak value within the SSRs. The SSRs always appear as monophasic negative responses.	μS
SPRs_Amp	Amplitude of the skin potential responses. Typically range from onset of the SPRs to the peak value within the SPRs. The SPRs can be either monophasic negative responses, biphasic responses (where an initial negative component is followed by a positive deflection), triphasic responses, where the positive limb of the biphasic response achieves a greater negativity than the initial negative wave, or monophasic positive in which no negative component is seen.	mV
SCRs_Tris	Time from onset of SCRs to peak SCRs. This is the time taken from the SCR onset to reach peak amplitude within the SCRs.	s
SPRET	Turning point of the SPRs relative to the SCR peak. SPRET is related to the relative time difference between the peaks of the SPR and SCR waveforms, which is due to the temporal distance of the SPR turning point (from negative to positive voltage direction) relative to the peak of the corresponding SCRs.	%

## Data Availability

Not applicable.
